# Stay Focused! The Effects of Internal and External Focus of Attention on Movement Automaticity in Patients with Stroke

**DOI:** 10.1371/journal.pone.0136917

**Published:** 2015-08-28

**Authors:** E. C. Kal, J. van der Kamp, H. Houdijk, E. Groet, C. A. M. van Bennekom, E. J. A. Scherder

**Affiliations:** 1 Research & Development, Heliomare Rehabilitation Centre, Wijk aan Zee, The Netherlands; 2 Faculty of Behavioural and Human Movement Sciences, VU University Amsterdam, Amsterdam, The Netherlands; 3 Institute of Human Performance, University of Hong Kong, Hong Kong; 4 Coronel Institute for Occupational and Environmental Health, Academic Medical Centre/University of Amsterdam, Amsterdam, The Netherlands; Centre de Neuroscience Cognitive, FRANCE

## Abstract

Dual-task performance is often impaired after stroke. This may be resolved by enhancing patients’ automaticity of movement. This study sets out to test the constrained action hypothesis, which holds that automaticity of movement is enhanced by triggering an external focus (on movement effects), rather than an internal focus (on movement execution). Thirty-nine individuals with chronic, unilateral stroke performed a one-leg-stepping task with both legs in single- and dual-task conditions. Attentional focus was manipulated with instructions. Motor performance (movement speed), movement automaticity (fluency of movement), and dual-task performance (dual-task costs) were assessed. The effects of focus on movement speed, single- and dual-task movement fluency, and dual-task costs were analysed with generalized estimating equations. Results showed that, overall, single-task performance was unaffected by focus (*p* = .341). Regarding movement fluency, no main effects of focus were found in single- or dual-task conditions (*p*’s ≥ .13). However, focus by leg interactions suggested that an external focus reduced movement fluency of the paretic leg compared to an internal focus (single-task conditions: *p* = .068; dual-task conditions: *p* = .084). An external focus also tended to result in inferior dual-task performance (β = -2.38, *p* = .065). Finally, a near-significant interaction (β = 2.36, *p* = .055) suggested that dual-task performance was more constrained by patients’ attentional capacity in external focus conditions. We conclude that, compared to an internal focus, an external focus did not result in more automated movements in chronic stroke patients. Contrary to expectations, trends were found for enhanced automaticity with an internal focus. These findings might be due to patients’ strong preference to use an internal focus in daily life. Future work needs to establish the more permanent effects of learning with different attentional foci on re-automating motor control after stroke.

## Introduction

Performing two or more tasks at the same time is integral to daily functioning. During the day, we frequently need to perform motor tasks like walking and grasping in combination with all sorts of cognitive (e.g., making a phone call, monitoring the traffic while crossing the street, memorizing a shopping list) or motor (e.g., carrying a tray) tasks. While healthy adults generally achieve this with ease, performing dual-tasks is often far more difficult for stroke patients, as their gait and balance often remain highly susceptible to interference from secondary cognitive task performance [1]. This increased dual-task interference may affect patients’ mobility, and has been linked to an increased risk of falling [2,3].

The challenge for clinicians therefore is to find ways to reduce patients’ dual-task interference. Successful dual-task performance depends on an individual’s working memory capacity [4]. Typically, it is assumed that during dual-tasking, each task consumes a share of working memory capacity. If the combined processing demands of two tasks exceed the capacity of working memory, dual-task interference will occur and performance on either or both these tasks will deteriorate [5]. Therefore, one way to improve dual-task performance is to reduce the demands placed on working memory, for instance, by increasing automaticity of movement.

Reducing the working memory processing demands of motor tasks may be achieved by manipulating the attentional focus of performers. Evidence from healthy adults shows that motor performance and learning are superior when performers focus on the outcome of their movements (i.e., an external focus) rather than on movement execution itself (i.e., an internal focus; for a review see [6]). According to the constrained action hypothesis [7] this is due to the fact that an external focus promotes automatic motor control, whereas an internal focus triggers conscious control of movement. In support of this hypothesis, an external focus has indeed been found to result in more automated movement execution in healthy participants, as evidenced by more efficient neuromuscular control (e.g., less muscular activity and co-contraction [8–10]) and more fluent and regular movement execution [11]. In line with the notion that enhanced movement automaticity reduces the demand for working memory resources [12, 13], an external focus also results in superior dual-task performance [11,14,15].

Observational studies have suggested that stroke patients primarily receive internally referenced instructions and feedback during rehabilitation therapy [16,17]. Also, many patients remain prone to use an internal focus to control their movements up to years after discharge [18]. If the evidence for the constrained action hypothesis obtained within healthy adults generalizes to the stroke population, one may hypothesize that patients’ and therapists’ predominant reliance on using an internal focus actually impedes patients’ automaticity of movement. As a result, this would not only impair their motor functioning, but possibly exacerbate dual-task interference as well. However, it is yet unclear whether the predictions of the constrained action hypothesis hold for stroke patients. In fact, results of the few studies that addressed the effects of attentional focus on single-task motor performance are ambiguous, with two studies reporting external focus [19,20] and one study reporting internal focus [21] to lead to superior upper extremity motor performance after stroke. The effects of attentional focus on patients’ automaticity of movement are even less well understood. Results of Fasoli et al. [20] and Durham et al. [19] showed that the deceleration phase of reaching was significantly shorter in duration when attention was focused externally compared to internally. The authors argued that these findings do suggest a reduction in on-line guidance during moving, but did not explicitly relate these findings to enhanced automaticity of movement. Also, neither study assessed whether an external focus results in superior dual-task performance after stroke.

Hence, the current study aimed to assess whether the constrained action hypothesis holds true for stroke patients by examining the immediate effects of internal and external attentional focus on motor performance in people with chronic (> 1 year), unilateral stroke. Patients performed a single-leg stepping task in isolation and in combination with two different cognitive dual-tasks. This experimental paradigm was chosen because it has been validated in an earlier study into attentional focus effects and dual-task interference in healthy adults [11]. If the constrained action hypothesis holds true for stroke patients, then they would demonstrate superior single-task leg-stepping performance (i.e., greater movement speed) with an external focus instruction compared to an internal focus instruction. In addition, we hypothesized that an external focus would result in enhanced movement automaticity, which would be evidenced by greater fluency of movement. Because enhanced movement automaticity captures less working memory capacity, we also anticipated an external focus instruction to result in reduced dual-task interference compared to an internal focus instruction. Finally, we explored whether individual differences (i.e., patients’ cognitive and motor capacities, and their inclination to use an internal focus in daily life) modified the (presumed differential) effects of attentional focus on single- and dual-task performance.

## Methods

### Participants

We recruited thirty-nine chronic stroke patients from three adult day care centers of Heliomare in the Netherlands between the 1^st^ of May 2013 and the 1^st^ of April 2014. Power analysis with G*power had shown that inclusion of at least 33 patients was necessary to be able to detect a small to moderate effect of focus on motor performance (based on repeated measures analysis of variance with an alpha-level of .05 and a power of .80). Inclusion criteria were as follows: 1) Unilateral, supratentatorial stroke confirmed by CT or MRI (obtained from patients’ records); 2) Time since injury > 1 year; 3) Capable of understanding instructions (i.e., able to perform the three-step command-item of the mini mental-state examination [22]); 4) Between 18 and 75 years old. All participants provided written informed consent.

### Ethics Statement

The study protocol was approved by the medical-ethical committee of the VU Medical Center in Amsterdam (VUMC protocol ID: 2012/463).

### Experimental Tasks

#### Motor Task

**Fig 1:**
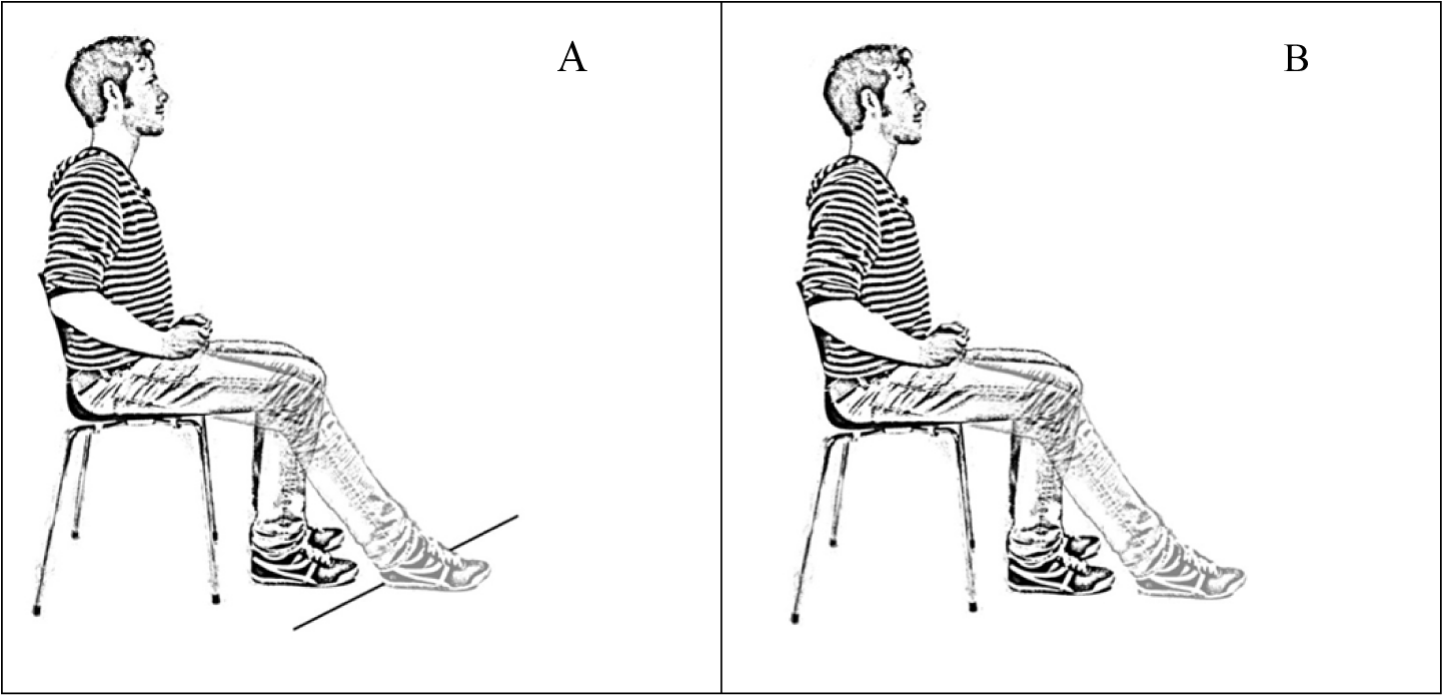
Motor Task. (A) In the external focus condition patients were instructed to focus on placing their foot in front of/behind a line that was taped on the floor. (B) In the internal focus condition patients were instructed to focus on flexing and extending their leg. Figure adapted with permission from Kal et al. [**11**].

The motor task was a single-leg-stepping task (Fig 1). Participants alternately flexed and extended their leg at a self-selected, comfortable pace for 60 seconds while seated [11]. Both legs were tested. In external focus conditions, a line was taped to the floor such that when participants placed their foot on the line their knee was flexed at an angle of 90 degrees (Fig 1, left panel). In the internal focus conditions this line was removed. Motor performance was defined as movement speed–i.e., the average absolute angular velocity in the anterior-posterior plane. Increases in comfortable pace were considered to reflect superior motor performance (analogous to tasks like the 10 meter timed walk test [23,24]. This leg-stepping paradigm was chosen because it is a highly controlled task that is easy and safe to perform, and because it enables us to separately investigate the effects of different foci on (relatively more automated) non-paretic and (relatively less automated) paretic leg performance. Finally, we have previously validated this paradigm in an earlier study into attentional focus effects within healthy participants [11].

Automaticity of leg-stepping performance was assessed by measuring the fluency of movement. The rationale is that as motor control becomes more automatic, movement fluency increases [25,26]. Movement fluency is typically operationalized as “jerk”, a measure that is derived from the minimal jerk model [27] and defined as the rate of change of acceleration of the moving limb. Thus, less jerky/more fluent movement execution is considered to reflect more automatic motor control.

#### Cognitive Tasks

Two different types of cognitive tasks were chosen: a letter fluency task (which is considered an executive function task) and an auditory reaction time task (taxing sustained attention). As reviewed by Al-Yahya et al. [28], reaction time tasks generally yield less dual-task interference than executive function tasks. Incorporating these two types of cognitive tasks thus allowed us to compare the effects of attentional focus on dual-task performance as a function of task difficulty. In addition, incorporating a reaction time task also allowed us to include patients who might have difficulties with the letter fluency task as a consequence of aphasia.

The letter fluency task required participants to name as many unique Dutch words as possible starting with a pre-specified letter within 1 minute. The outcome variable was the total number of words. Nine letters with a similar level of difficulty [29] were chosen: D-A-T-K-O-M-P-G-R.

For the auditory reaction time task (ARTT), participants were presented with 18 auditory stimuli: 9 target stimuli (i.e., car horn) and 9 non-target stimuli (either the sound of a bell, a barking dog, or a whistle). Participants were required to react as fast as possible by saying “yes” whenever the target stimulus was presented, but had to ignore the non-target stimuli. Each stimulus was presented for 300 ms at 3-second intervals, with a time delay of -750 ms, -375 ms, 0 ms, +375 ms, or +750 ms to prevent anticipation. Order of stimuli and time delays were randomized. The dependent variable was reaction time in ms (for the correct responses).

#### Neuropsychological & Motor Assessments

Patients’ level of education [30] and score on the Dutch version of the MMSE [22] served as general measures of cognitive capacity. Furthermore, specific tests for executive functioning [31–33], working memory [34–35], and attention [31,32,36] were administered (see Table 1). Raw scores were corrected for patients’ educational level and age by calculating Z-scores. For the executive function and attention domains, Z-scores of subtests were averaged, yielding one Z-score for each domain (see Table 1).

**Table 1:** Cognitive domains and associated neuropsychological tests.

Cognitive domain	Test	Alternative for aphasics	Outcome parameter
*Executive Function*	D-KEFS TMT-switching condition (divided attention) [31]	Color Trails Test-switching condition (divided attention) [32]	Time to complete (s)
	Tower of London (planning abilities) [33]	N/A	# moves needed to complete whole test
*Working Memory*	WAIS–letter/number sequencing [34]	WAIS—Symbol Span [34]	# correct sequences
*Attention*	D2-concentration test [36]	N/A	CP-score
	D-KEFS TMT—number sequencing condition [31]	Color Trails Test—number sequencing condition [32]	Time to complete (s)

NB: For aphasic patients, the Color Trails Test and WAIS Symbol Span were administered as alternatives for the D-KEFS and WAIS letter/number sequencing tests. CP-score = concentration performance score; TMT = trail making test; WAIS = Wechsler Adult Intelligence Scale;

# = number of; N/A = not applicable.

Motor capacity of the most-affected leg was assessed with the lower extremity subscales of the Fügl-Meyer Assessment [37] and Motricity index [38].

Finally, to assess patients’ preference to monitor and control their movements with an internal focus in daily life, patients filled out the Dutch version of the Movement-Specific Reinvestment Scale (MSRS) [39,40]. This self-report scale includes 10 items. Five items form the subscale “Movement Self-Consciousness”, and reflect the degree to which someone feels self-conscious about his/her style of movement (i.e., “I am concerned about what people think about me when I am moving”). The other 5 items belong to the “Conscious Motor Processing” subscale, and reflects one’s inclination to consciously control movements in daily life (“I try to think about my movements when I carry them out”). Items are scored on a 6-point Likert scale ranging from 0 (strongly disagree) to 5 (strongly agree). Hence, scores range from 0–25 for each subscale, and between 0–50 for the whole scale. Higher reinvestment scores suggest a stronger preference to explicitly monitor (Movement Self-Consciousness) and control (Conscious Motor Processing) movements in daily life [41], and hence, suggest a stronger preference to focus internally.

### Procedure

Measurements were performed on three occasions, separated by at least 24 hours. Pilot testing revealed the whole protocol to be too fatiguing and time-consuming to be completed within one measurement occasion.

**Fig 2:**
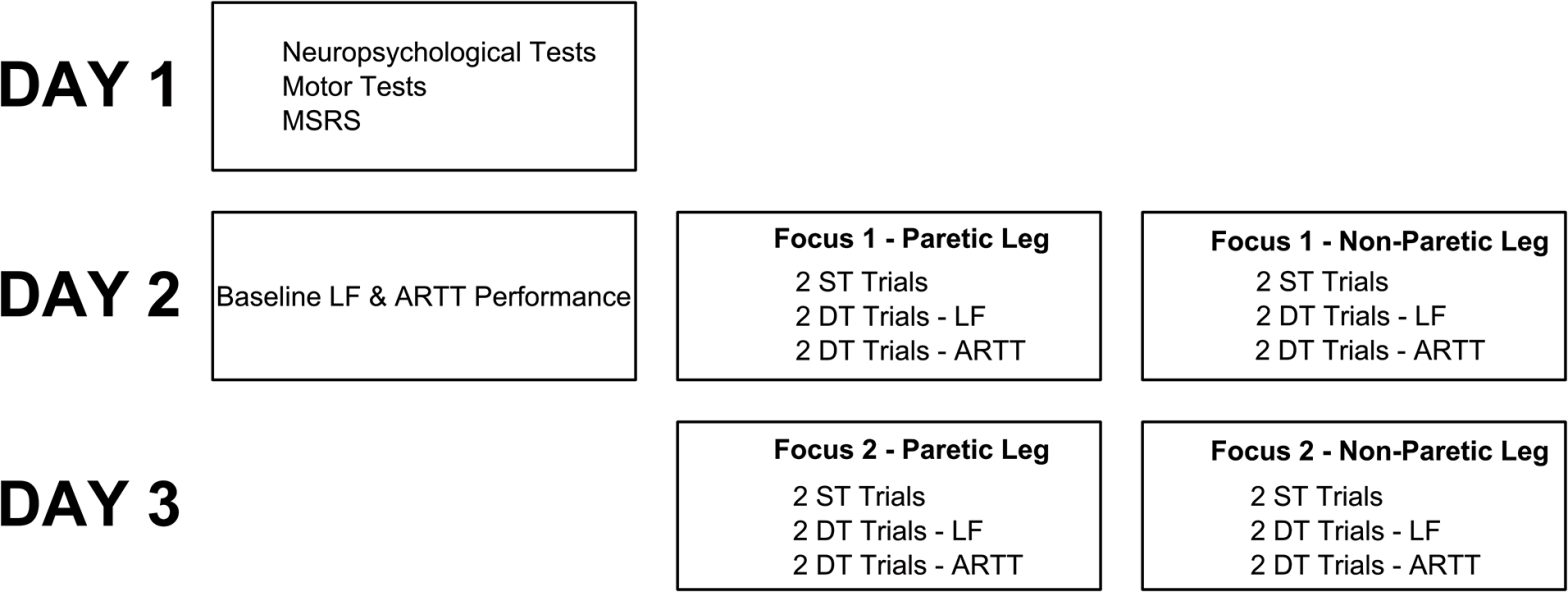
Measurement protocol. NB: ARTT = Auditory Reaction Time Task; ST = single-task; DT = Dual-task.

On the first measurement day, the neuropsychological tests, the Dutch MSRS, and tests of motor capacity were administered. On the second measurement day, single-task performance on the letter fluency task and ARTT was assessed. Next, participants performed two blocks (first with one leg, then with the other) with the same attentional focus consisting of six 60-second trials: two single-task trials, two letter fluency dual-task trials, and two ARTT dual-task trials. For dual-task trials, patients were instructed to prioritize the motor task. Prior to the start of each trial, attentional focus was instructed. Internal focus instructions were to focus on “alternately flexing and extending the leg”, whereas external focus instructions were to focus on “alternately placing the foot in front of and behind the line” [11]. During trials, instructions were (briefly) repeated every 20 seconds to ensure compliance. Trials were separated by two minutes of rest. On the third measurement day, this procedure was repeated with the other focus. Also, single-task performance on the ARTT and letter fluency task was assessed again. The order of focus (i.e., external versus internal focus) was counterbalanced across participants. Participants always performed the two single-task trials first. The order of the motor-cognitive dual-task conditions (leg-stepping task + ARTT versus leg-stepping task + letter fluency) and legs (affected versus non-affected) was counterbalanced across participants. Fig 2 summarizes the experimental procedure.

### Equipment and Data Collection

Seismic tri-axial hybrid accelerometers (DynaPort-MiniMod; McRoberts B.V., The Hague, The Netherlands) were used to measure the acceleration and angular velocity of the lower legs during the leg movement task. Accelerometers were attached to the tibia, approximately halfway an imaginary line from the lateral epicondyle to the lateral malleolus. The xyz-coordinate system of the accelerometer was defined such that the x-axis pointed forward (i.e., in the anterior-posterior plane), the y-axis pointed sideward (i.e., in the medio-lateral plane), and the z-axis pointed upward (i.e., in the transversal plane) when the knee was flexed 90 degrees. Data was stored at an internal SD card at 100 Hz.

Focus instructions and auditory stimuli were presented with customized software (Mixcraft 6; Acoustica Inc; CA; USA) via a headset. Number of words named (letter fluency task) and responses on the ARTT were recorded with a directional microphone, and sampled at 10000 Hz using customized LabVIEW software (National Instruments; Austin; Texas). All experimental trials were recorded with a video-camera with sound recording.

### Data Analysis

Accelerometer data and ARTT data were analysed with customized Matlab programs (Mathworks, Natick MA, USA). As each condition was measured twice (e.g., two single-task trials for each task, two letter fluency dual-task trials, two ARTT dual-task trials), average values were calculated for each condition.

To assess motor performance, the angular velocity in the mediolateral plane was filtered with a bidirectional, low-pass Butterworth filter (cut-off frequency: 5 Hz), rectified, and averaged over the whole 60 seconds of each trial, yielding the average movement speed per trial in degrees per second. Movement fluency was operationalized as the dimensionless jerk [27]. It is imperative to use the dimensionless jerk rather than the raw jerk, as raw jerk values are biased by differences in movement duration and amplitude [27]. Dimensionless jerk was determined as in our previous study [11]. For each flexion-extension movement cycle the resultant acceleration was calculated, and normalized (divided by its mean). Next, the derivative of the normalized resultant acceleration was obtained, yielding the mean rectified jerk. Then, to obtain a dimensionless measure, the mean rectified jerk values were multiplied with the duration of the flexion-extension cycle. Finally, calculating the mean of dimensionless jerk values across all movement cycles yielded the average dimensionless jerk for the whole trial.

Letter fluency performance was defined as the number of words per trial. Task performance was scored offline from video recordings by an independent neuropsychologist who was blind to the study goal. ARTT performance was assessed by determining the median difference (in ms) between target stimuli and associated responses for each trial. Dual-task performance was operationalized by calculating dual-task costs (DTCs; [42]) for motor and cognitive tasks (see Eq 1). A positive DTC reflects a deterioration in performance in dual-task conditions.

DTC=(STperformance–DTperformance)/STPerformancex100% (1)

### Statistics

All statistical analyses were executed using SPSS version 20.0. The effects of attentional focus on single-task movement speed and movement fluency were analysed with two separate generalized estimating equation (GEE) analyses. GEE is a type of regression analysis that corrects for the dependency of repeated measurements. We chose an exchangeable working correlation matrix to define dependency amongst measurements. movement speed or movement fluency were the dependent variables, while focus (external vs. internal) and leg (paretic vs. non-paretic) were predictors.

Before comparing dual-task performance between conditions, we first checked whether significant dual-task interference occurred. Holm-Bonferroni [43] corrected paired-samples t-tests were conducted to test whether DTCs were significantly different from zero. Subsequently, the effect of attentional focus on dual-task costs was assessed with GEE, with DTCs as the dependent variable, and focus (external vs. internal), leg (paretic vs. non-paretic), source (of DTCs; motor vs. cognitive) and type of dual-task (letter fluency vs. ARTT) as predictors. A similar GEE was then conducted with movement fluency as dependent variable to assess whether dual-task movement fluency differed as a function of focus, leg, and type of dual-task.

For all the above GEE analyses, significance of interactions between the main predictors (i.e., focus, leg, source, and type of dual-task) was assessed. The first, preliminary GEE model included all possible interactions. Using a backward approach, the (least contributing) interaction term was removed in turn, such that only near-significant (*p* < .10) interaction terms were retained in the final GEE model.

Finally, we explored whether the effect of focus on single- and dual-task performance was modified by cognitive capacity (executive function, working memory, or attention domain z-scores), motor capacity (Fügl-Meyer and Motricity Index), and/or patients’ preferences for using an internal focus (reinvestment-scores). These variables were added to the single- and dual-task performance GEE-models in turn. Effect modifiers were identified if they significantly interacted with the predictor focus.

## Results

All 39 patients completed the experiment (see Table 2 for characteristics). Worthy of note, 7 patients were incapable of performing the motor task with their paretic leg. Five other patients could not complete the letter fluency test, due to severe expressive aphasia. One patient showed extreme jerk scores (> 3 SDs above group mean) and was therefore excluded from the jerk analyses. In all, 27 patients performed the whole protocol (assessment of both legs in both motor-cognitive dual-task conditions), and 32 performed the whole protocol minus the letter fluency task.

**Table 2:** Patient characteristics.

Group Characteristics	*Mean ± SD*
*n*	39
*Age in years ± SD*	62.62 ± 8.6
*Female/Male*	17/22
**Lesion location: Left/Right**	20/19
**Lesion aetiology**	
*Haemorrhage*	12
*Infarction*	27
**Time since stroke (months)**	113 ± 87
**Aphasia: Yes/No**	13/26
**Cognitive Capacity**	
*Education level* ^*a*^ *(0–6)*	4.15 ± 0.8
*MMSE (0–30)*	28 ± 2.2
*Executive Function (Z-score)*	-1.05 ± 1.1
*Working Memory (Z-score)*	-0.76 ± 0.9
*Attention (Z-score)*	-1.36 ± 0.9
**Motor Capacity (of lower extremity)**	
*Fügl-Meyer (0–28)*	19.3 ± 5.8
*Motricity Index (%)*	63.1 ± 18.7
**Movement-Specific Reinvestment Scale (0–50)**	31.8 ± 7.2
*Movement Self-Consciousness (0–25)*	13.7 ± 5.7
*Conscious Motor Processing (0–25)*	18.1 ± 3.5

^a^ Education level is based on the international standard classification of education [30]

### Single-Task Results

#### Effect of Focus of Attention on Single-Task Motor Performance

**Fig 3:**
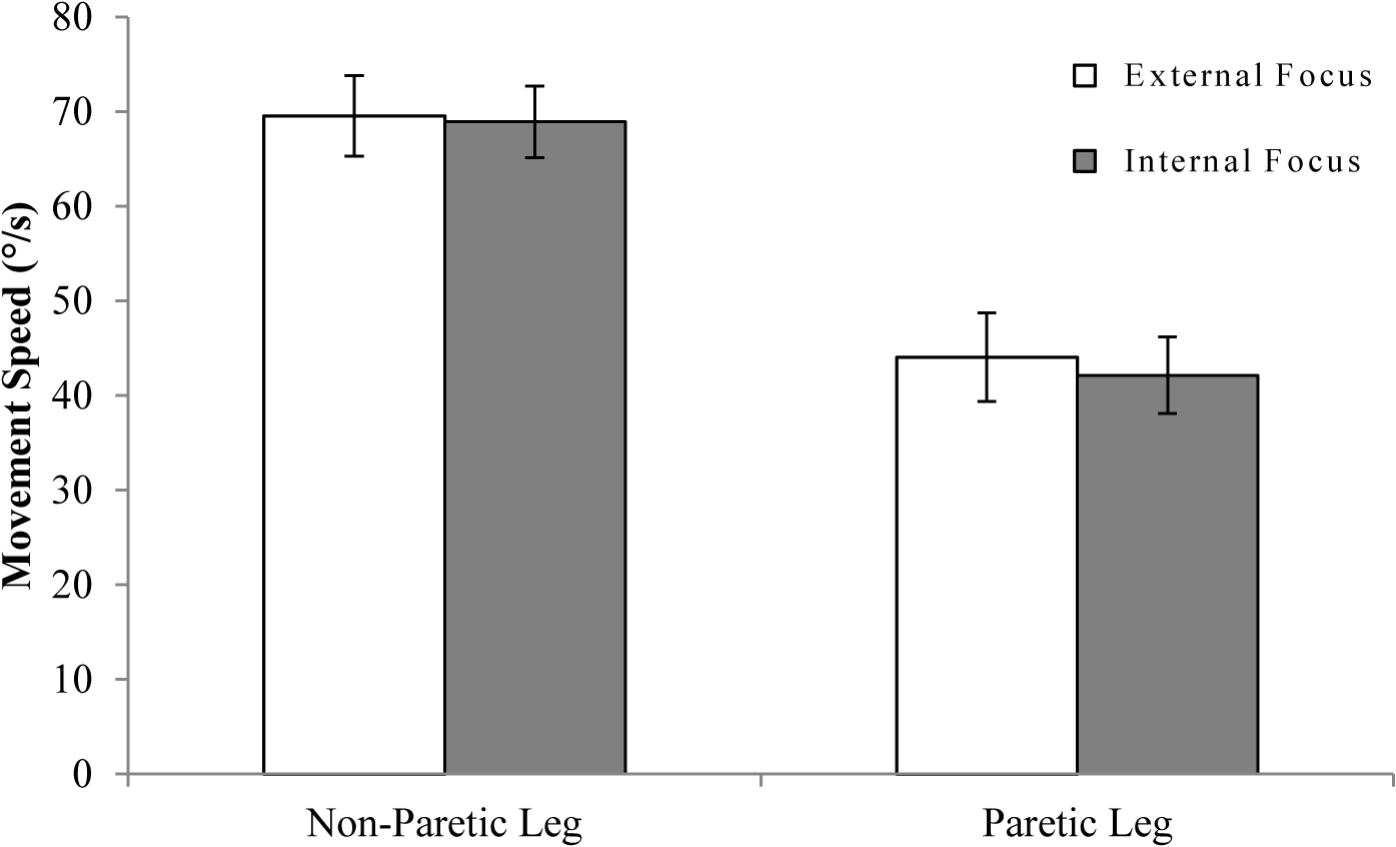
Single-task movement speed. Movement speed is expressed in degrees per second ± SE.

Single-task motor performance results are depicted in Fig 3. GEE analysis (Table 3) revealed no significant differences in movement speed between internal and external focus conditions (*p* = .341), but higher speeds in non-paretic compared to paretic leg movements (*p* < .001). As no significant interaction was found between focus and leg (*p* = .387), this interaction term was left out of the final single-task GEE model (see Table 3).

**Table 3:** Summary of results of GEE analyses of single- and dual-task performance.

	*Wald χ* ^*2*^	*Beta*	*p*	*95% CI of Beta*
**Single Task Movement Speed**				
Focus *(Internal vs*. *External)*	0.91	-1.92	.341	[-3.7, 0.9]
Leg *(Non-Paretic vs*. *Paretic)*	47.26	28.17	**< .001**	[20.1, 36.2]
**Dual-Task Costs**				
Focus *(Internal vs*. *External)*	3.40	-2.38	.*065*	[-4.9, 0.1]
Leg *(Non-Paretic vs*. *Paretic)*	69.35	-8.68	**< .001**	[-10.7, -6.6]
Source of DTCs *(Motor vs*. *Cognitive)*	65.60	-18.85	**< .001**	[-23.4, -14.3]
Type of Dual-Task *(ARTT vs*. *Letter Fluency)*	4.20	-4.50	**.040**	[-8.8, -0.2]

NB: Significant p-values are emphasized, while near-significant p-values are in italics.

Subsequent effect modification analyses revealed that the effect of focus on single-task performance was not modified by patients’ cognitive capacity or Motricity Index scores (all: *p*’s ≥ .2). However, patients’ Fügl-Meyer scores (Wald χ^2^ = 2.99, β = -.38, *p* = .084, 95% *CI* = [-.81, .05]) and reinvestment scores (Wald χ^2^ = 6.56, β = .40, *p* = .010, 95% *CI* = [.09, .70]) did modify the effect of focus. That is, patients with higher Fügl-Meyer scores showed larger improvements in leg-stepping speed in external focus conditions (β = 2.32) than in internal focus conditions (β = 1.93). Also, patients with higher reinvestment scores showed larger reductions in leg-stepping speed in external focus conditions (β = -.81) than in internal focus conditions (β = -.41). Closer inspection of MSRS-subscale scores revealed this effect to be most pronounced for Movement Self-Consciousness scores (β = .49, *p* = .018), and less so for Conscious Motor Processing scores (β = .53, *p* = .15). Combined, these findings suggest that patients with more pronounced motor impairments and stronger reinvestment tendencies benefit more from internal focus instructions than from external focus instructions (and vice versa).

#### Effect of Focus of Attention on Single-Task Movement Fluency

**Fig 4:**
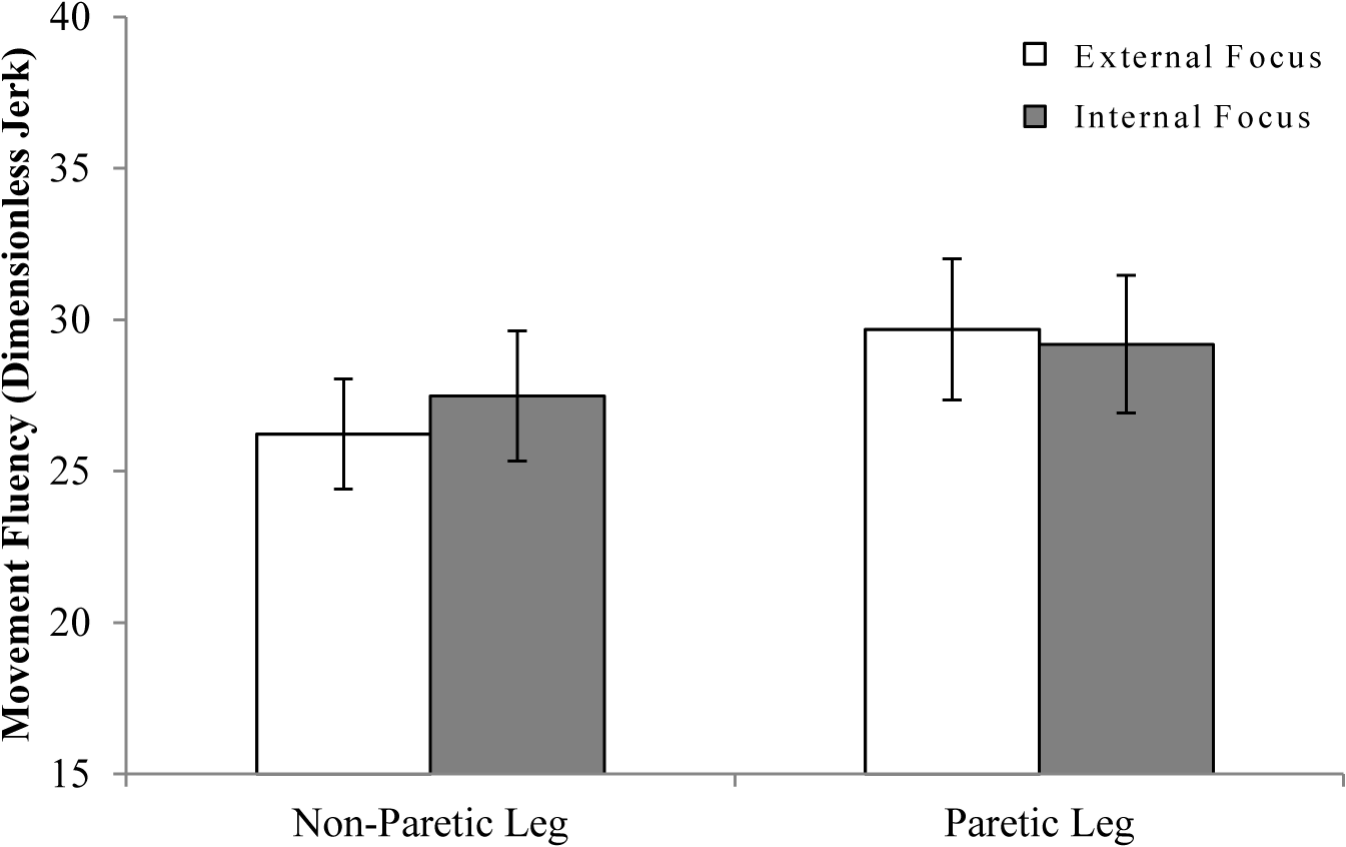
Single-task movement fluency. Movement fluency is expressed in dimensionless jerk ± SE, with lower values indicating more fluent movement execution.

Fig 4 shows results of movement fluency during single-task conditions, while Table 4 lists results of the corresponding GEE analysis. Movement fluency did not differ as a function of attentional focus (*p* = .644). Non-paretic leg movements were significantly more fluent than paretic leg movements (*p* = .011). However, the near-significant interaction between focus and leg (*p* = .068) indicated that this difference in fluency between legs was more pronounced in external focus conditions (*p* = .062) than in internal focus conditions (*p* = .380).

**Table 4:** Summary of results of GEE analyses of movement fluency.

	*Wald χ* ^*2*^	*Beta*	*p*	*95% CI of Beta*
**Single Task Movement Fluency**				
Focus *(Internal vs*. *External)*	.214	-.49	.644	[-2.6, 1.6]
Leg *(Non-Paretic vs*. *Paretic)*	6.52	-3.65	**.011**	[-6.4,-.8]
Focus x Leg Interaction*	3.33		.*068*	
*Internal Focus x Paretic Leg*	4.52	3.16	**.034**	[0.2, 6.1]
*Internal Focus x Non-Paretic Leg*	2.52	1.26	.112	[-0.3, 2.8]
*External Focus x Paretic Leg*	6.52	3.65	**.011**	[0.8, 6.4]
**Dual-Task Movement Fluency**				
Focus *(Internal vs*. *External)*	2.27	-.99	.132	[-2.3, 0.3]
Leg *(Non-Paretic vs*. *Paretic)*	5.58	-3.04	**.018**	[-5.6,-.5]
Type of Dual-Task *(ARTT vs*. *Letter Fluency)*	3.14	-1.34	.*076*	[-2.8, 0.1]
Focus x Leg Interaction*	2.99		.*084*	
*Internal Focus x Paretic Leg*	2.30	2.05	.130	[-0.6, 4.7]
*Internal Focus x Non-Paretic Leg*	.07	-0.13	.795	[-1.1, 0.8]
*External Focus x Paretic Leg*	7.20	3.90	**.007**	[1.1, 6.7]

NB: Significant p-values are emphasized, while near-significant p-values are in italics.

* = for the interaction terms, External Focus x Non-Paretic Leg served as reference.

#### Dual-Task Costs

**Fig 5:**
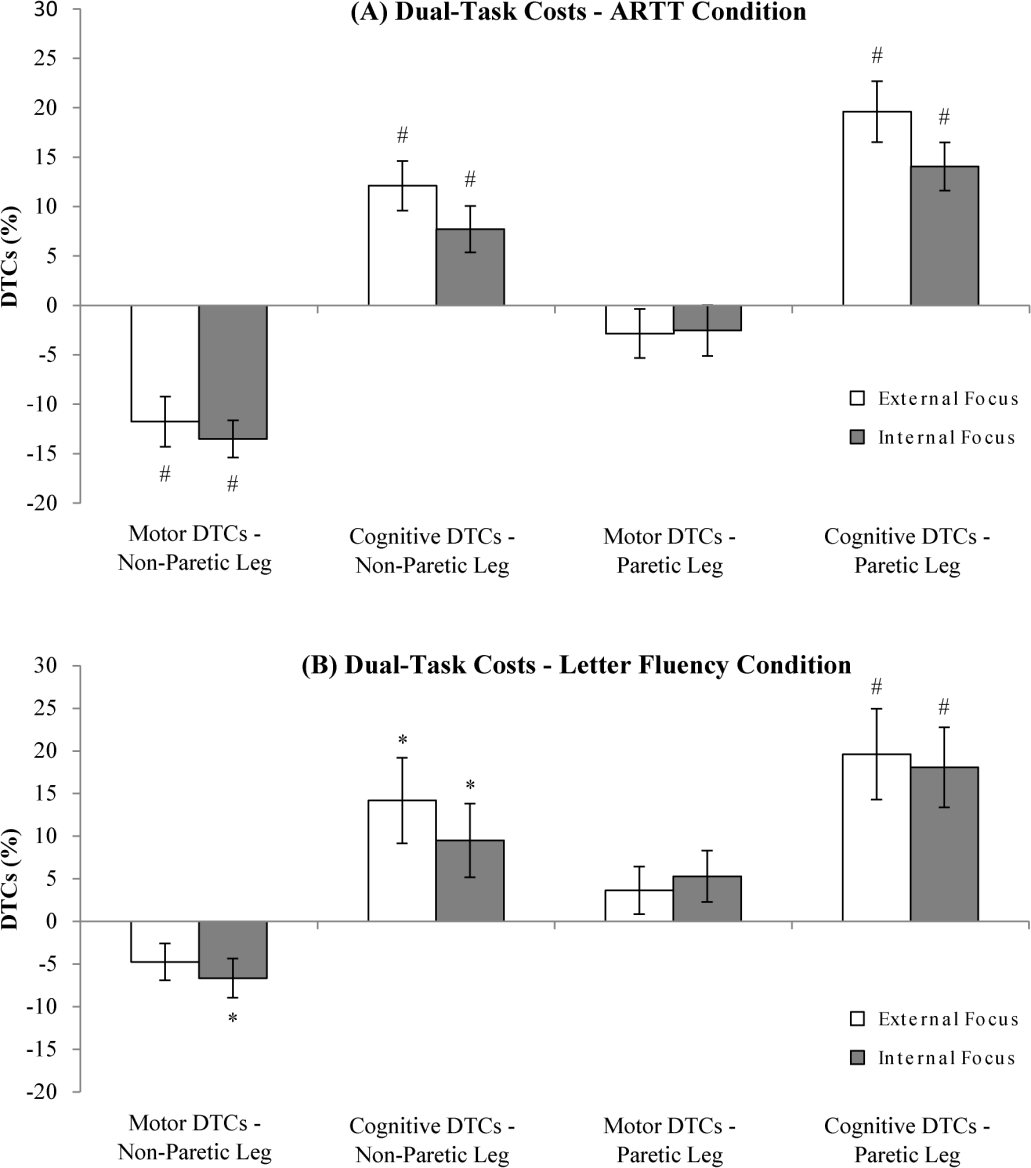
Average motor and cognitive dual-task costs. (A) Dual-task costs for the ARTT dual-task conditions. (B) Dual-task costs for the letter fluency dual-task conditions. Dual-task costs are expressed in percentages ± SE. Positive dual-task costs indicate deteriorated performance compared to single-task conditions. Striped bars represent external focus conditions, solid bars indicate internal focus conditions. Dual-task costs that significantly differ from zero (i.e., single-task performance) are marked with an * (p < .05) or with an # (p < .01). NB: DTC = dual-task cost.

At baseline, stroke patients on average listed 9.9 words (± 3.9) on the letter fluency task, and responded within 539 ms (± 163) on the target stimulus in the auditory reaction time task (ARTT). First, we assessed whether significant dual-task interference occurred when these tasks were simultaneously performed with the leg-stepping task (see Fig 5 for a summary of dual-task costs). To this end, we determined whether DTCs significantly differed from zero–i.e., single-task performance–using Holm-Bonferroni t-tests. Motor DTCs for the auditory reaction time task were significantly lower than zero for the non-paretic leg (*t’s* (38) > 4.6, *p* < .01, *d* > 1.5) but not for the paretic leg (*t’s* (31) < 1.1, *p* > .3, *d* < .41). This indicated that the non-paretic leg moved faster in ARTT dual-task conditions than in single-task conditions. For the letter fluency dual-task conditions, no significant motor DTCs were found (*t’s*(26–33) ≤ 2.2, *p’s* ≥ .17, *d’s* ≤ .77), with the exception that significant negative DTCs were evident for the non-paretic leg in internal focus conditions (*t*(33) = 2.9, *p =* .04, *d* = 1.0). With regard to cognitive DTCs, significant positive DTCs were noted for both the ARTT and letter fluency dual-task conditions (*t’s* (26–38) > 2.2, *p’s* < .05, *d’s* > 0.77; see Fig 5). In sum, although motor performance was not disrupted by dual-tasking, cognitive task performance deteriorated.

#### Effect of Focus of Attention on Dual-Task Costs

Having established that significant dual-task interference occurred (especially for the cognitive tasks), we subsequently assessed whether DTCs differed as a function of focus, leg, source, and type of dual-task. The corresponding GEE-analysis revealed a trend towards significance for focus (*p* = .065), and significant effects for leg (*p* < .001), source (*p* < .001), and type of dual-task (*p* = .040), but no interaction effects (all *p*’s > .2; Table 3). The near-significant effect of focus was due to an internal focus generally leaning toward lower DTCs than an external focus. Also, significantly lower DTCs were noted for the non-paretic compared to the paretic leg conditions, for the motor compared to the cognitive task conditions, and for the ARTT compared to letter fluency task conditions.

Subsequent effect modification analyses revealed that focus did not significantly interact with motor capacity, executive function, working memory, or reinvestment scores (all *p*’s > .3). However, we did find a near-significant interaction between focus and attention domain scores (Wald χ^2^ = 3.69, β = 2.36, *p* = .055, 95% *CI* = [-.05, 4.76]): Better attentional capacity tended to reduce dual-task costs in external focus conditions (β = -2.98) more than in internal focus conditions (β = -.62).

#### Effect of Focus of Attention on Dual-Task Movement Fluency

**Fig 6:**
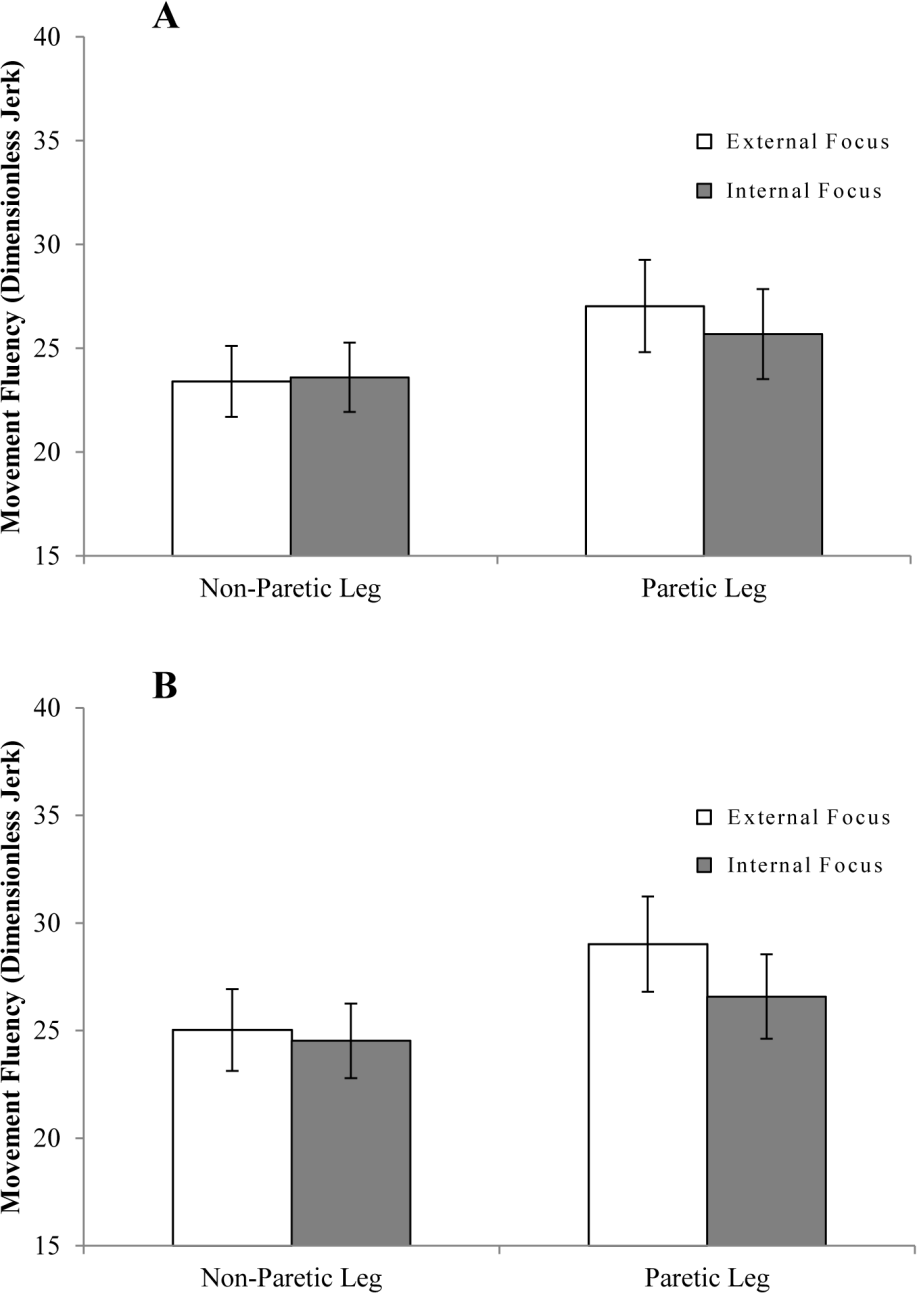
Dual-task movement fluency. (A) Movement fluency results for the ARTT dual-task conditions. (B) Movement fluency results for the letter fluency dual-task conditions. Movement fluency is expressed in dimensionless jerk ± SE. Lower jerk values indicate greater movement fluency.

Fig 6 shows fluency of movement during dual-task conditions. Overall, movement fluency was similar in internal and external focus conditions (*p* = .132; Table 4), but greater for the non-paretic leg than for the paretic leg (*p* = .018). However, similar to the analysis of single-task movement fluency, a near-significant focus by leg interaction was found (*p* = .084). This suggested that movement fluency only differed between legs when attention was focused externally (*p* = .043) but not when attention was focused internally (*p* = .282). Finally, movement execution tended to be more fluent in ARTT dual-task conditions than in letter fluency dual-task conditions (p = .076).

## Discussion

This study set out to test the constrained action hypothesis within chronic stroke patients. Specifically, we examined the prediction that–compared to an internal focus–an external focus acutely enhances chronic stroke patients’ motor performance by promoting more automatic motor control. To this end, we compared the effect of external and internal focus instructions on patients’ leg stepping speed, as well as on a kinematic proxy of automaticity: fluency of movement. Finally, as more automatic movements should place a lower demand on working memory resources, we also assessed whether external focus instructions enhanced dual-task performance compared to internally referenced instructions.

### Effect of Focus on Motor Perfermance, Automaticity of Movement, and Dual-Task Performance of Stroke patient

Single-task movement speed remained stable in the face of different attentional focus instructions, regardless which leg was used. This sharply contrasts the numerous studies that have found an external focus to lead to superior motor performance in healthy adults [6–8]. The present results are especially at odds with those of our previous study in which we used an identical leg-stepping paradigm, and found that healthy adults demonstrate superior single-task leg-stepping speed with external focus instructions compared to internal focus instructions [11]. Our present results thus add to the heterogeneity of earlier findings regarding the effects of attentional focus on motor behavior after stroke [19–21]. At the very least, this suggests that for chronic stroke patients as a group, an external focus does not acutely benefit single-task motor performance compared to an internal one.

The analyses of fluency of movement and dual-task performance may provide clues as to why the motor performance benefits obtained within healthy adults do not seem to uniformly generalize to the stroke population. Congruent with the single-task movement speed results–but contrary to hypothesized–an external focus did not result in greater movement fluency than an internal focus, neither in single- nor in dual-task conditions. Focus by leg interactions suggested a reverse pattern, with an external focus reducing movement fluency of the paretic leg. These findings seem in line with the analysis of dual-task performance, which also failed to show a benefit of an external focus of attention. Rather, DTCs tended to be higher when patients focused externally, and patients’ attentional capacity tended to constrain dual-task performance in external but not internal focus conditions. Combined, these findings tentatively suggest that an external focus was more reliant on attentional functioning (and hence: less automatic) than an internal focus. Again, as for the single-task results, these findings sharply contrast those of our previous study, in which healthy adults showed superior movement fluency and dual-task performance with external focus instructions [11].

In sum, the constrained action hypothesis’ predictions were not confirmed within a group of chronic stroke patients: Compared to an internal focus, an external focus of attention did not acutely benefit motor performance, enhance fluency of movement, or reduce dual-task interference. Weak but consistent findings of reduced automaticity with an external focus might imply that external focus instructions can have a negative effect on automaticity of movement and dual-task performance of stroke patients.

### Effect of Attentional Focus on Automaticity–Modulating Role of Focus Familiarity and Attentional Capacity

What could possibly explain the unexpected lack of enhanced–and trends toward reduced–automaticity with an external focus? A possible explanation stems from Maurer and Munzert [44], who showed that the effect of the *direction* of attentional focus (i.e., internal vs. external) on motor performance can be confounded by the performer’s preference for either type of focus (see also [45]). In two experiments, healthy adults performed best (on a golf-putting and on a basketball free throw task) when they were instructed to use the attentional focus they were most familiar with, regardless whether this constituted an external or internal focus. To explain these findings, the authors proposed that ‘Frequently used attentional strategies may become integrated parts of the skill and no longer impact on automated skill execution’ (p. 737). By contrast, adopting a non-familiar focus is highly attention-demanding, and hence disrupts automated motor performance. A similar phenomenon may in part explain the results of our study. The high reinvestment scores of our patient group (Table 2) suggest that the majority of patients was prone to habitually adopt an internal focus of attention. Building on Maurer and Munzert’s results, we hypothesize that focusing internally may thus have been a more familiar, less attention-demanding strategy for these patients than adopting an external focus. This hypothesis is in line with the finding that for patients with high reinvestment scores single-task leg-stepping speed was enhanced by internal rather than external focus instructions. Furthermore, this hypothesis would explain why adopting an external focus especially reduced fluency of paretic leg movements (especially if one assumes that patients are most inclined to focus internally when moving their most-affected leg), why attentional capacity seemed more important for dual-task performance under an external focus of attention, and hence, why patients performed worst at dual-tasking in external focus conditions.

Admittedly, the hypothesized role of preferred focus would be more strongly supported if patients’ reinvestment scores had also directly modulated the effect of attentional focus on dual-task performance. The fact that they did not might partly be due to the fact that reinvestment scores clustered at the top end of the scale range, with 75% of patients scoring 25 points or higher. Future research may explicitly address the presumed role of focus preference in stroke patients and healthy adults in more detail. Possibly, these studies may also use measures that more directly assess (the strength of) individual focus preferences, for instance by having performers rate the mental effort required to adhere to different focus instructions [44].

### Dual-Task Performance–Effects of Legs, Type of Dual-Task, and Task Prioritization

A final note concerns the difference in dual-task performance that emerged as a function of leg, type of dual-task condition, and source of costs (motor vs. cognitive). The fact that the ARTT yielded less dual-task interference than the letter fluency task fits the results of a recent meta-analysis [28]. The observation that dual-tasking primarily affected cognitive task performance indicates that patients complied with the instruction to prioritize motor performance. The differences between the paretic and non-paretic leg are of more interest, though. As expected, clear-cut differences were evident between legs in terms of dual-task performance; patients were more proficient at dual-tasking with their non-paretic leg than with their paretic leg. In the apparently easiest (ARTT) condition, non-paretic leg movement speed even increased compared to single-task conditions. These findings are in agreement with reports that distracting attention away from movement execution can benefit motor performance, as long as the motor skill is sufficiently automated and the secondary task is relatively easy [46,47]. Taken together, it seems that stroke patients may invest a superfluous amount of attention into their (otherwise relatively automated) non-paretic leg movements, even to the extent that it constrained their single-task performance. Patients’ strategy to consciously control their movements–although likely intended to deal with the motor impairments of their paretic leg–thus also seemed to affect motor control of their non-paretic leg.

### Limitations and Implications for Future Research

The present study yields new insights and triggers new questions regarding the effects of attentional focus on (automaticity of) motor performance post-stroke. Its immediate implications for clinical practice are limited, though, for several reasons. For one, this study addressed acute performance effects, not motor learning (i.e., the long term retention of (re-)acquired motor skills). Second–although it allowed us to investigate the effects of focus for both legs separately–the experimental leg-stepping task seems of limited functional relevance. It remains to be seen whether the results obtained with this highly controlled, relatively simple task generalize to more complex, clinically relevant motor tasks like walking. Still, the validity of this motor task seems supported by the fact that both Fügl-Meyer and Motricity Index scores significantly predicted performance on this task (β_FM_ = 2.32, β_MI_ = .058; both *p*’s < .01). Third, the stroke group in the present study mostly consisted of stroke patients who had suffered brain damage a relatively long time ago (almost 10 years on average), and who have all been involved in rehabilitative physical therapy in which they likely received a lot of internally referenced instructions and feedback [16,17]. For greater clinical relevance, future studies should compare the long-term effects of different attentional foci on re-acquiring and re-automating clinically meaningful motor skills (e.g., gait or postural control) already in the clinical/inpatient phase of stroke. Furthermore, it is not unlikely that differences between different foci of attention average out on a group level, due to the large heterogeneity in cognitive and motor functioning within the stroke population. Therefore, an important venue for future research is to more specifically explore the modulatory role of individual patient characteristics. In this regard, the present study suggests that patients’ motor capacity, focus preferences, and attentional capacity may be of interest. Further exploration of these issues is required in order to establish whether (and for whom) attentional focus instructions can be used to facilitate motor learning after stroke.

Finally, a general limitation of studies into the effect of attentional focus instructions on motor performance is that one can never be absolutely certain that participants complied with instructions. In this experiment, we tried to maximize compliance in several ways. First, before the start of the internal/external focus block patients were asked to repeat the instructed focus. Second, during each trial, instructions were briefly repeated at 20 and 40 seconds. Third, the instructions used in this study have been found to reliably induce external and internal foci of attention in our earlier study [11]. The fact that patients complied with the instruction to prioritize motor performance over cognitive task performance further strengthens our confidence that they also complied with attentional focus instructions.

## Conclusion

In conclusion, the present study’s results did not confirm the constrained action hypothesis’ predictions within a chronic stroke population. Relative to an internal focus, an external focus did not directly enhance patients’ motor performance, fluency of movement, or dual-task performance. Although effects were weak, it might be that an internal focus facilitates automatic motor control after stroke, possibly due to patients’ pronounced inclination to consciously control their movements in daily life.

## Supporting Information

S1 Table Experimental Data. Supplementary tables containing the individual data used for the analyses in our manuscript. Individual patient characteristics (Table A; Demographics), individual data regarding movement speed (Table B; Single-Task Movement Speed), movement fluency (Tables C and D; Single-task Movement Fluency & Dual-task Movement Fluency), and dual-task costs (Table E; Dual-Task Costs).
(XLSX)
